# Explorative visual analytics on interval-based genomic data and their metadata

**DOI:** 10.1186/s12859-017-1945-9

**Published:** 2017-12-04

**Authors:** Vahid Jalili, Matteo Matteucci, Marco Masseroli, Stefano Ceri

**Affiliations:** Dipartimento di Elettronica, Informazione e Bioingegneria, Politecnico di Milano, Milano, 20133 Italy

**Keywords:** Genomic data analysis, exploration, visualization, Interactive and visual analytics, Comparative evaluation, Next Generation Sequencing

## Abstract

**Background:**

With the wide-spreading of public repositories of NGS processed data, the availability of user-friendly and effective tools for data exploration, analysis and visualization is becoming very relevant. These tools enable interactive analytics, an exploratory approach for the seamless “sense-making” of data through on-the-fly integration of analysis and visualization phases, suggested not only for evaluating processing results, but also for designing and adapting NGS data analysis pipelines.

**Results:**

This paper presents abstractions for supporting the early analysis of NGS processed data and their implementation in an associated tool, named GenoMetric Space Explorer (GeMSE). This tool serves the needs of the GenoMetric Query Language, an innovative cloud-based system for computing complex queries over heterogeneous processed data. It can also be used starting from any text files in standard BED, BroadPeak, NarrowPeak, GTF, or general tab-delimited format, containing numerical features of genomic regions; metadata can be provided as text files in tab-delimited attribute-value format. GeMSE allows interactive analytics, consisting of on-the-fly cycling among steps of data exploration, analysis and visualization that help biologists and bioinformaticians in making sense of heterogeneous genomic datasets. By means of an explorative interaction support, users can trace past activities and quickly recover their results, seamlessly going backward and forward in the analysis steps and comparative visualizations of heatmaps.

**Conclusions:**

GeMSE effective application and practical usefulness is demonstrated through significant use cases of biological interest. GeMSE is available at http://www.bioinformatics.deib.polimi.it/GeMSE/, and its source code is available at https://github.com/Genometric/GeMSE under GPLv3 open-source license.

## Background

High-throughput sequencing technologies generate high amounts of genomic, epigenomic and transcriptomic data regarding multiple genomes in different conditions. Complex pipelines are used for selecting high-quality sequenced raw data, aligning them to a reference genome, and then calling specific features on the aligned data, such as DNA mutations, transcription factor bindings, histone modifications, DNA methylations, gene expressions [1, 2]. Thanks to large international consortia (e.g., Encyclopedia of DNA Elements (ENCODE) [3], Roadmap Epigenomics [4], The Cancer Genome Atlas (TCGA) [5], and the 1000 Genomes Project [6]), such data are organized within open repositories, which provide easy access to raw and processed datasets. The availability of these datasets is reshaping modern biology: researchers can complement their own experimental datasets with a large body of public data and knowledge, and can derive relevant results which are just based upon secondary analysis of open data.

GenoMetric Query Language (GMQL) [7] is an innovative cloud-based system to efficiently compute arbitrarily complex queries over heterogeneous processed datasets, taking into account both genomic region features and sample global characteristics (i.e., metadata). GMQL queries apply to genomic datasets of Next Generation Sequencing (NGS) processed data to extract interesting data samples and their genomic regions and metadata; such valuable GMQL output needs further data exploration and analysis to support biological interpretation of results.

This paper presents a rich set of abstractions for data analysis, exploration and visualization, and their implementation in an associated tool, named GenoMetric Space Explorer (GeMSE); GeMSE supports primitives for data explorations spanning from *select*, *sort*, and *discretize*, to *clustering*, and *pattern extraction*. GeMSE seamlessly manages metadata together with genomic region data and shows them aggregated for any of the result clustering patterns. GeMSE leverages on GMQL as its back-end tertiary data retrieval framework, but can be used on any text files in standard BED (Browser Extensible Data), BroadPeak, NarrowPeak, GTF (General Transfer Format), or general tab-delimited format, containing data regarding features of genomic regions; metadata can also be provided as text files, in tab-delimited attribute-value format.

Genomic data visualization builds on two orthogonal concepts: genome browsing and quantitative visualization. A genome browser, pioneered by Artemis [8] and popularized by the University of California at Santa Cruz (UCSC) Genome Browser [9], is commonly used for looking at genome features within a given portion of the genome. In the realm of quantitative visualizations, clustering techniques and heatmaps (proposed outside biology) were used by Eisen and colleagues [10] for the evaluation of microarray gene expression data; they have been implemented in some stand-alone tools (e.g., GENE-E [11]) and they are supported in many statistical software, including Matlab, Mathematica and R/Bioconductor [12], as well as scripting languages such as Python. Lately, they have been applied to NGS data, and implemented within a few tools specifically devoted to such data (e.g., seqMINER [13], ngs.plot [14], or MicroScope [15]). These tools are mainly designed to be used on NGS raw or aligned data; unless they are executed on very powerful servers, they can handle only a few data files at a time, limiting the possibility of quickly comparing multiple conditions and datasets simultaneously.

GeMSE can be regarded as enabler of *interactive analytics* (IA), a promising exploratory approach for the seamless “sense-making” of data through on-the-fly integration of analysis and visualization tools. Interactive analysis is suggested not only for evaluating processing results, but also for designing and adapting NGS data analysis pipelines. Remarkably different results could be produced with slightly different parameter settings of data production pipelines (e.g., for feature calling); choosing a “correct” parameter setting commonly breaks down to a difficult cycle of repeatedly tweaking parameters, re-running the analysis, and visually inspecting the results. Tweaking the parameters of the tools used for data generation is context-specific and could consist of tweaking parameters of GMQL scripts or Galaxy workflows [16]; other examples of IA frameworks include Cytosplore [17], focused on mass cytometry data for immune systems cellular composition studies, or Trackster [18], which leverages Galaxy’s comprehensive data analysis framework (spanning from primary to tertiary analysis).

Data exploration is well supported by application suites such as Mathlab, Mathematica, Maple or SageMath (in Python), or scripting languages such as Python, R, Perl, or even shell scripting; however, not everyone has the required scripting/coding ability. GeMSE enables data exploration using intuitive visual interfaces for everyone, without need for any scripting, making data exploration seamless.

A key component of explorative data analysis, is to be able to perform actions in a **non-sequential** and **repeatable** way. To enable such data exploration, GeMSE adapts a state-space graph model, where nodes/states are the data and transition are the actions performed on the data. Users can choose any node, and perform any number of actions on a node (hence creating a new node), while all nodes are efficiently cached in memory, enabling the creation of (theoretically) an unlimited number of states. In general, every action by the user generates a new state/node, which can then be used in subsequent analyses, downloaded, or visualized. Nodes are immutable, i.e., once a node is generated, it cannot be changed (changes happen as new nodes). A key advantage of this feature is that if the user makes a mistake or wants to experiment with different parameter settings, he/she can always go back to the original data.

## Implementation

Datasets in GMQL consist of one or more items, called *samples*, each of them associated with one experimental condition; each sample, in turn, consists of data and metadata. Data are genomic regions, expressing the result of a calling process that extracts genomic features (e.g., DNA mutations, gene expression scores, peaks of binding enrichment, epigenetic modifications) from measured (epi)genome signals. Metadata are attribute-value pairs expressing arbitrary properties of samples (e.g., the related tissue or cell-line, the technology used to obtain it, the experimental method applied; if the sample is human, it may include phenotypical information, such as the donor’s sex, age and disease status).

### Genometric space

**Fig. 1:**
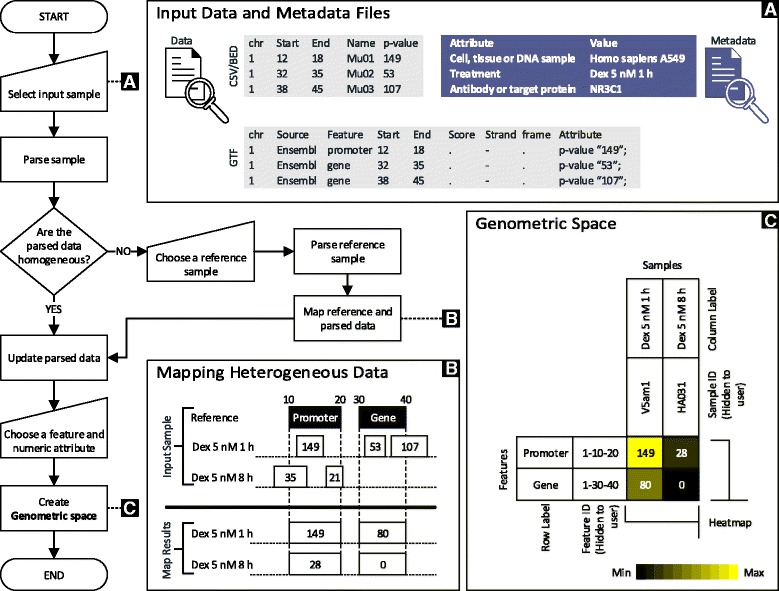
Importing data and building genometric space. A sample is represented with two files: data and metadata. To enable exploring samples using both quantitative and descriptive aspects, GeMSE loads both files. The flowchart shows the flow of loading the files. Panel **A** shows an example of data (in CSV/BED and GTF format), and metadata of a sample. Panel **B** depicts an example of mapping heterogeneous samples using a reference sample (multiple values are aggregated using *average* function). Panel **C** illustrates a genometric space, and how data are organized to form it. Columns (samples) and rows (regions) have column and row IDs which are respectively sample and regions IDs in parsed data. The IDs are hidden to the user, and are used to label columns and rows with any attribute that the user chooses (e.g., the *treatment* and *feature name* attributes for labeling columns and rows respectively)

A *genometric space* is produced by a specific GMQL operation, called MAP [7], which applies to two datasets, denoted as *reference* and *experiment* (see panel b on Fig. 1):

- The *reference dataset* consists of a single sample; it typically includes genomic regions corresponding to genes or exons, representing the coding portions of the genome, or transcription regulatory regions; however, the reference sample can be an arbitrary set of regions from the genome, possibly extracted by means of GMQL queries.
- The *experiment dataset* consists of multiple, possibly heterogeneous, samples, each constituted by multiple regions (similar to heterogeneous tracks that can be observed on a genome browser); experiment samples can be produced by different sources, while we expect each experiment sample to be produced by a single source.

The MAP operation produces a matrix structure, called *genometric space*, where each row is associated with a reference region, each column refers to a sample, and each matrix entry is computed by means of an aggregate function applied to the values of a selected attribute of the experiment regions of the sample that overlap the reference region (see panel c on Fig. 1). Formally:

- The MAP operation applies to a reference sample *R* and to several experiment samples *S*_*j*_, and has two parameters: an attribute *A* of the regions of *S*_*j*_ and an aggregate function *G*.
- The result of the MAP operation is a matrix *M*, whose entries *m*_*i,j*_ are each built from the region *r*_*i*_ of the reference and the sample *S*_*j*_ of the experiment dataset by considering all regions *r*_*k,j*_ of the *S*_*j*_ sample having a nonempty intersection with *r*_*i*_, then considering the bag (i.e., set) *B*_*i,j*_ of all the values *v*_*k,j*_ that the attribute *A* gets for the *r*_*k,j*_ regions, and then applying the aggregate function *G* to *B*_*i,j*_.

We support the classic aggregate functions COUNT, MIN, MAX, SUM, AVERAGE, and MEDIAN; COUNT is used to count the number of experiment sample regions intersecting a reference region, and requires no indication of a specific attribute. The 2×2 matrix in panel c on Fig. 1 represents 2 genomic regions and 2 experiment samples; values are ((149,28),(80,0)). The matrix is organized in GeMSE with the reference regions as rows and the experiment samples as columns ; this choice is preferred because there are typically many more regions than experiments.

When GeMSE is used in pipeline with GMQL, it reads the output of a GMQL MAP operation directly; when instead GeMSE is used as a stand-alone tool, it starts by applying a MAP operation to the reference and experiment samples specified by the user (see flowchart, panel a, and panel b on Fig. 1). Input region data can be read as formatted according to the standard BED, BroadPeak, NarrowPeak, or GTF formats, or in the form of a general BED-like tab-delimited format. Required fields of each region are *chromosome* (i.e., *chr*), *start*, and *end*, as in the BED format. Additional fields are considered as referenced by the correspondent input column header; e.g., GTF files in addition contain the fields *source*, *feature* (i.e., feature name), *score*, *strand*, *frame*, and a *group* field which is a text string containing a set of attribute-value pairs separated by a single space. Metadata can also be provided as separate tab-delimited text files, having the same name as the sample file to which they refer to, and an extension “.meta”, storing items in a pair of fields, respectively called *attribute* and *value*. The flowchart in Fig. 1 shows that files of heterogeneous formats can be given in input to GeMSE.

### Interactive data exploration model

**Fig. 2:**
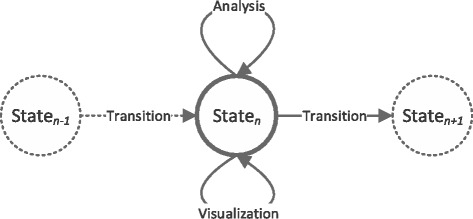
The data exploration model of GeMSE

GeMSE data exploration consists of three iterative phases, illustrated on Fig. 2 and explained as it follows:

- *Transition*, where a transformation function is applied on a genometric space resulting in a new genometric space.
- *Analysis*, where a genometric space is analyzed using data analysis functions (e.g., pattern analysis, or statistical inference).
- *Visualization*, where a genometric space is visualized (e.g., on heatmaps or graph views).

In GeMSE, genometric spaces are immutable and independent from each other; in other words, once a genometric space is created, it cannot be changed. Therefore, to enable data exploration, GeMSE organizes genometric spaces on a state-transition tree, explained in the following section. The genometric space transitions and analysis are explained the subsequent sections.

### State-transition tree

**Fig. 3:**
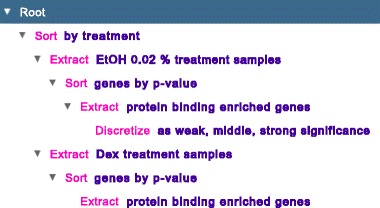
An example of GeMSE State-Transition Tree; it represents the use-cases illustrated in the demonstration and discussion section of the paper

Tracking multiple transformations of genometric spaces is crucial for data exploration. GeMSE tracks such transitions in a graph data structure called *State-Transition Tree* (STT), whose nodes represent different genometric spaces and whose edges represent the transformations between genometric spaces (e.g., see Fig. 3). From any data exploration state, one can view the related genometric space, visualizing it as a table or a heatmap, and also explore contained patterns (e.g., see Fig. 7, where the heatmaps labeled A1-A5 and the associated pattern exploration refer to the first sequence of nodes on Fig. 3). STT visualization facilitates data exploration state examination and a trial-and-error approach.

GeMSE stores nodes and edges of STT in memory. However, keeping all the nodes in memory is not an efficient practice, specially if the STT and genometric spaces are considerably large. Therefore, GeMSE implements the *least recently used* caching algorithm [19]. Accordingly, GeMSE stores the first data exploration state (i.e., the root of the STT), the genometric space of the *n* most recent states (with the *n* value being user modifiable), and the transitions of all the states. Least recently used states are removed from the memory, and if needed they are reconstructed. This is done first by recursively traversing the STT from the node to be reconstructed to the closest cached parent node; then, once the closest cached parent node is determined, the requested node is reconstructed by applying the stored transitions from the closest cached parent node to the requested one. Given that clustering is computationally expensive, dendrograms, i.e., cluster hierarchical structures, are always kept in memory to prevent cluster reconstruction.

### State transitions

A state transition takes a state and some arguments as input, and generates a new state as output. In our case, a state transition is a data transformation performed during data exploration, and a state represents the explored data, in case resulting from one of such transitions. The general data transformations most useful in data exploration, which we implemented in GeMSE, are: Extract, Rewrite, Discretize, Sort, Cluster, and Bi-Cluster. In what follows, we give a semi-formal description of each of such state transitions as a genometric space transformation. It is important to note that these operations are specified in a very simple way by using the GeMSE tool, with an easy-to-use graphical interface that prompts, for each transformation, the parameters to be interactively entered.

#### Extract

This transformation extracts a sub-space *S* ^′^ of a genometric space *S*, given two ranges of columns and rows. Let [*C*_*l*_,*C*_*r*_) and [*R*_*u*_,*R*_*d*_) denote ranges for columns (with left and right bounds) and rows (with up and down bounds), respectively (inclusive lower-bound, exclusive higher-bound); the transformation is defined as follows:

$$ \begin{aligned} S^{\prime} = {} & \texttt{Extract} (\\ & [C_{l}, C_{r}), \\ & [R_{u}, R_{d})) \\ & S \end{aligned} $$

**Fig. 4:**
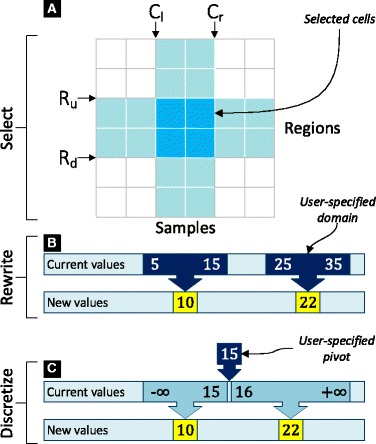
An example of Select (panel **A**), Rewrite (panel **B**), and Discretize (panel **C**) transformations

After an Extract operation, the new state in the STT holds a new genometric space *S* ^′^, which is a subset of the input state *S* (represented in light blue in panel a on Fig. 4). The data and metadata of the selected samples/rows are not changed, while the data and metadata of excluded samples/regions are discarded at the new state.

#### Rewrite

This transformation maps the values of an input genometric space *S* into new values in a new genometric space *S* ^′^; if only a portion of *S* where to apply the transformation is selected, all the other values of *S* outside the selected portion remain unchanged, and the dimensions of *S* ^′^ are not modified with respect to those of *S*. The values of *S* are mapped conditionally; the values of cells [*C*_*l*_,*C*_*r*_), [*R*_*u*_,*R*_*d*_) are mapped to a constant *V*, or log*n* transformed (user-defined *n*), if the values are within the [*V*_min_,*V*_max_]_*i*_ range. Several ranges may be used in the same Rewrite transformation, provided that the ranges do not overlap (e.g., see panel b on Fig. 4). Rewrite is a discrete mapping, such that the ranges not necessarily cover all the values in the input genometric space; the excluded values remain intact. Each value is changed based on the range that it falls in, e.g., {[*V*_min_,*V*_max_]_1_→*V*_1_,[*V*_min_,*V*_max_]_2_→*V*_2_,… }. The transformation is defined as follows:

$$ \begin{aligned} S^{\prime} = {} & \texttt{Rewrite} (\\ & [C_{l}, C_{r}), \\ & [R_{u}, R_{d}), \\ & ([V_{\text{min}}, V_{\text{max}}], [V \vert \log_{n}])^{+}) \\ & S \end{aligned} $$

#### Discretize

This transformation maps all the values of an input genometric space *S* to new values in a new genometric space *S* ^′^, in case selecting only a portion of *S* where to apply the transformation. The difference between the Rewrite and Discretize transformations is that Rewrite is a discrete mapping of values, whereas Discretize is a contiguous mapping; accordingly, the transformation ranges are specified differently (see panels b and c on Fig. 4). In Rewrite, users explicitly define the ranges [*V*_min_,*V*_max_]_*i*_, which are user-defined independent ranges and not necessarily contiguous. Conversely, in Discretize, users define transformation ranges implicitly, by using break values (pivots) [*V*_pivot_]_*i*_, based on which the transformation ranges are determined automatically. For instance, referring to panel c on Fig. 4, suppose the Discretize transformation operates on Natural numbers, and takes the pivot value 15 and the new values 10 and 22; then, the Discretize transformation automatically defines the ranges (−*∞*,15] and [16,+*∞*), and maps the values in these two ranges to 10 and 22, respectively. Note that when this transformation operates on real numbers, the ranges around a pivot value *V*_pivot_ are as (−*∞*,*V*_pivot_] and (*V*_pivot_,+*∞*).

The Discretize transformation has also a *NoChange* option, which indicates that the values within a given range should not be changed. The transformation is defined as follows:

$$ \begin{aligned} S^{\prime} = {} & \texttt{Discretize} (\\ & [C_{l}, C_{r}), \\ & [R_{u}, R_{d}), \\ & (V_{\text{pivot}}, [V_{\mathrm{b}} \,\vert\, \texttt{NoChange}], [V_{\mathrm{a}} \,\vert\, \texttt{NoChange}])^{+}) \\ & S \end{aligned} $$

where *V*_b_ and *V*_a_ are the values with which the values before and after the *V*_*pivot*_ value are respectively replaced.

#### Sort

This transformation sorts the rows or columns (*R*|*C*) of an input genometric space *S* in ascending/descending order, based on the values of a list of region attributes (e.g., count, *p*-value), or of sample metadata (e.g., antibody target, disease), and stores the ordered result in a new genometric space *S* ^′^. The transformation is defined as follows:

$$ \begin{aligned} S^{\prime} = {} & \texttt{Sort} (\\ & [\texttt{R} \,\vert\, \texttt{C}], \\ & [\texttt{ASCENDING} \,\vert\, \texttt{DESCENDING}], \\ & [(\text{Region Attribute})^{+} \,\vert\, (\text{Sample Metadata})^{+}]) \\ & S \end{aligned} $$

#### Cluster

This transformation executes the clustering of either rows or columns (*R* | *C*) of an input genometric space *S*, and produces as output a clustered genometric space *S* ^′^, as well as a dendrogram (hierarchical description of the various clustering steps) and a heatmap, that plots the genometric space sorted based on the dendrogram. The Cluster transformation performs agglomerative hierarchical clustering by single, average, or complete linkage (*SINGLE* | *AVERAGE* | *COMPLETE*), using distance and correlation metrics; GeMSE implements *Euclidean* (EU), *Manhattan* (MA), *Earth Movers* (EA), *Chebyshev* (CH), and *Canberra* (CA) distance metrics, and *Pearson correlation* (PE) metrics. The transformation occurs by first producing the clustering dendrogram, and then using the dendrogram for sorting the genometric space rows (regions) or columns (samples). The transformation is defined as follows:

$$ \begin{aligned} S^{\prime} = {} & \texttt{Cluster} (\\ & [\texttt{R} \,\vert\, \texttt{C}], \\ & [\texttt{SINGLE} \,\vert\, \texttt{AVERAGE} \,\vert\, \texttt{COMPLETE}], \\ & [{\tt EU} \,\vert\, {\tt MA} \,\vert\, {\tt EA} \,\vert\, {\tt CH} \,\vert\, {\tt CA} \,\vert\, {\tt PE}]) \\ & S \end{aligned} $$

#### Bi-cluster

This transformation clusters both rows and columns simultaneously of an input genometric space *S*. To implement it in GeMSE, we used the R package *hclust* [20] (see “Availability and requirements” section), which performs bi-clustering by complete linkage (*COMPLETE*) using the *Euclidean* (*EU*) distance metrics. GeMSE automatically creates a script to be executed in R, then runs the script, and finally imports the generated result (i.e., a heatmap in *.png* format). Thus, the Bi-Cluster transformation in GeMSE does not generate a state that can be used for further transitions, since GeMSE has access to the clustering output of R as a heatmap only. The generated heatmap (i.e., output genometric space representation) is therefore a leaf node of the state-transition tree. The transformation is defined as follows:

$$ \begin{aligned} S^{\prime} = {} & \texttt{Bi-Cluster} (\\ & [\texttt{COMPLETE}]), \\ & [{\tt EU}]) \\ & S \end{aligned} $$

GeMSE supports other transformations performed by means of R packages; some of them (e.g., *gplots* [21]) require first a normalization of the distances of the clustering dendrogram from the leaves to the root; then, the updated dendrogram is exported to R in *Newick tree* format [22], along with the genometric space on which to apply it and the R script to be run. All these transformations with R-based implementations produce only the heatmap representation of the output genometric space; thus, in the state transition tree all of them generate a leaf node only, which is not usable for further transitions.

### State analysis

An analysis function takes a state, and executes data analysis function on it. GeMSE implements two commonly used class of data analysis functions: pattern extraction, and statistical inference (e.g., statistical hypothesis testing, or principal component analysis), briefly described in the following sections.

#### Pattern extraction

A relevant task in data exploration concerns with the identification of patterns in the data, and their association with specific data aspects (e.g., biological features, supporting biological interpretation of the results).

**Fig. 5:**
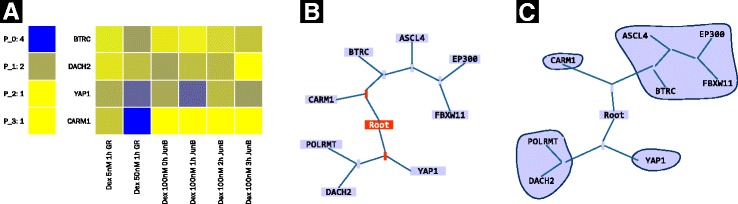
Patterns exploration options: **A** heatmap, where each row represents a pattern and is labeled by the name of one of the elements of the pattern, and each column shows the counts of each of the patterns. **B** radial graph, where each node represents a vector (pattern analysis input), and edges are the relations between the nodes. Nodes colored *red*, are the nodes above the dendrogram cut, and nodes colored *purple* are below dendrogram cut; hence all the nodes colored *purple* after a *red* node, belong to the same pattern. **C** Force-directed graph, where nodes belonging to the same pattern are aggregated

Within a data matrix (i.e., genometric space), a pattern can be defined as an ensemble of feature values associated with a group of rows/columns which are similar based on such values. These patterns can be discovered through the Cluster data transformation implemented in GeMSE, by using either distance (e.g., *Euclidean* or *Manhattan* distance) or correlation (e.g., *Pearson correlation*) metrics between vectors of rows/columns containing such feature values; these vectors are clustered hierarchically, and patterns are extracted by cutting the clustering dendrogram at a given height. By doing so, the nearest (most similar) vectors of rows/columns are grouped together, unveiling a pattern. Patterns can then be explored in GeMSE by means of:

- *Heatmaps*, which effectively visualize each pattern (e.g., panel a on Fig. 5 and panel A5pc on Fig. 7).
- *Radial graph* [23], where nodes are the pattern analysis vectors (columns or rows of the genometric space), and edges are the relations between vectors. The visualization is interactive, it enforces a radial ordering of the nodes, while keeps a user-selected node at the center. Additionally, if selected by the user, it can color nodes differently, based on the pattern analysis result (see panel b on Fig. 5).
- *Force-directed graph* [23]; it is an interactive visualization forcing a graph view, which can aggregate nodes belonging to the same pattern (user-selected, see panel c on Fig. 5).
- *Vectors forming the pattern*, displayed in forms of *heatmaps* (e.g., panels A2p0, A2p1, and A2p2 on Fig. 7), or *tabular views* of vector values or metadata (e.g., the table on Fig. 7).
- *Metadata counts*, representing the aggregated occurrences of each metadata attribute-value pair in each pattern (e.g., the table on Fig. 9); they facilitate the identification of common/exclusive metadata within each pattern, and the interpretation of patterns based on such metadata.

#### Number of clusters

A key aspect in the described pattern extraction strategy is the choice of where cutting the dendrogram so as to identify an ideal number of patterns. GeMSE can suggest the best number of clusters; it does so by taking advantage of the clustering dendrogram produced by the Cluster data transformation, and by using the *Elbow* method [24]. This method compares the sum of squared distances between clusters for different number of clusters, plotted against the number of clusters; the optimal number of clusters is determined by identifying an “elbow” in the plot. To identify it, we first determine the total variance of the distances between the children of all nodes in the clustering dendrogram (i.e., between all clusters). Then, we calculate the variance percentage as the variance of the distances between the children of the nodes in the dendrogram (i.e., between clusters) at different dendrogram cutting heights (i.e., for different number of clusters), divided by the total variance. Finally, we compare the slope of two consecutive points in the plot (i.e., the variation of variance percentage for two consecutive dendrogram cutting heights, that is for two consecutive numbers of clusters): an “elbow” is where the difference of slopes between consecutive points is maximum (see Fig. 8). The pseudocode of the method is given in Algorithm 1.



Several other methods exist to determine the best number of clusters, based on gap statistic [25], or on “stopping rules” [26], or exploiting the Direction Division Partitioning principle [27] (i.e., stopping partitioning when centroid scatter value exceeds the maximum cluster scatter value at any node in the clustering dendrogram). Other methods are based on maximizing the distance between patterns and relative closeness [28], or on information criterion approaches - such as Akaike information criterion [29], Bayesian information criterion [30], or Deviance information criterion [30]. Note that no method performs always well; particularly, the Elbow method does not work well if the data are not very clustered. The GeMSE user can always interactively define the number of clusters to consider.

#### Statistical inference

Samples (columns) or regions (rows) of a genometric space can represent results of different hypothesis testing (e.g., DNA-protein binding significance); hence, GeMSE implements commonly used statistical inference methods to test (null and alternative) hypothesis, deduce properties, and evaluate correlation and dependencies between samples or regions. The methods for statistical inference implemented in GeMSE follow in the following two classes:

- *Statistical hypothesis testing*: GeMSE allows the hypothesis testing based on the following statistics computed for a genometric space: t-statistic, one-sample and two-tailed t-test, two-sided t-test. GeMSE also evaluates if the null hypothesis can be rejected accordint to a given *α* confidence, *p*-value, approximated degree of freedom, and homoscedasticity.
- *Covariance and correlation*: To spot correlation and dependencies, GeMSE allows performing covariance, Pearson product-moment correlation coefficient, and principle component analysis among genometric space row or columns.

GeMSE allows users to interactively choose a genometric space and an analysis to be performed, and to setup the required parameters; then, it visualizes data as single values (e.g., *p*-values) or plots, using scatter plots or heatmaps.

## Results

We demonstrate the effective application and practical usefulness of GeMSE using 33 NGS Chromatin Immunoprecipitation sequencing (ChIP-seq) datasets from the *Homo sapiens* A549 immortalized cell line (an epithelial cell line derived from lung carcinoma tissue) [31], downloaded from ENCODE [3].

### Datasets

The datasets used are summarized on Table 1; they cover various types of experiments, spanning different treatments and targeting various DNA-binding proteins.

- Some datasets belong to studies assessing the effect of treatments with Dexamethasone (Dex) on the DNA-binding enrichment profile of different proteins, including the treatments (a) with various doses of Dex (500 pM, 5 nM, and 50 nM) on NR3C1, a glucocorticoid receptor protein (see rows 1-3 on Tables 1 and 2), or (b) with 100 nM of Dex on transcription factor jun-B for multiple durations (30 m, 0 h, 1 h, 2 h, 3 h, 4 h, 5 h, 7 h, 8 h, and 10 h; see rows 4-13 on Tables1 and 2), or (c) with 100 nM of Dex for 1 h on different transcription factors (FOXA1, POLR2A, USF1; see rows 14-16 on Tables 1 and 2).
- Some other datasets belong to studies assessing the effect of 1 h treatment with 0.02 % of Ethanol (EtOH) on different DNA-binding proteins (e.g., ATF-3, CTCF, jun-D; see rows 17-29 on Tables 1 and 2), or to studies assessing the activity of DNA-binding proteins under no treatment (see rows 30-33 on Tables 1 and 2).

**Table 1:** Datasets of human A549 immortalized cell line used for GeMSE demonstration

#	Treatment	Dose	Duration	Antibody target	Replicates
1	Dexamethasone	500 pM	1 h	NR3C1	∙∙
2	Dexamethasone	5 nM	1 h	NR3C1	∙∙
3	Dexamethasone	50 nM	1 h	NR3C1	∙∙
4	Dexamethasone	100 nM	30 m	JUNB	∙∙
5	Dexamethasone	100 nM	0 h	JUNB	∙∙
6	Dexamethasone	100 nM	1 h	JUNB	∙∙
7	Dexamethasone	100 nM	2 h	JUNB	∙∙
8	Dexamethasone	100 nM	3 h	JUNB	∙∙∙
9	Dexamethasone	100 nM	4 h	JUNB	∙∙∙
10	Dexamethasone	100 nM	5 h	JUNB	∙∙∙
11	Dexamethasone	100 nM	7 h	JUNB	∙∙∙
12	Dexamethasone	100 nM	8 h	JUNB	∙∙∙
13	Dexamethasone	100 nM	10 h	JUNB	∙∙
14	Dexamethasone	100 nM	1 h	FOXA1	∙∙
15	Dexamethasone	100 nM	1 h	POLR2A	∙∙
16	Dexamethasone	100 nM	1 h	USF1	∙∙
17	Ethanol	0.02 %	1 h	ATF3	∙∙∙
18	Ethanol	0.02 %	1 h	BCL3	∙∙
19	Ethanol	0.02 %	1 h	CTCF	∙∙
20	Ethanol	0.02 %	1 h	EP300	∙∙
21	Ethanol	0.02 %	1 h	GABPA	∙∙
22	Ethanol	0.02 %	1 h	JUND	∙∙
23	Ethanol	0.02 %	1 h	POLR2A	∙∙
24	Ethanol	0.02 %	1 h	REST	∙∙
25	Ethanol	0.02 %	1 h	SIN3A	∙∙
26	Ethanol	0.02 %	1 h	SIX5	∙∙
27	Ethanol	0.02 %	1 h	TAF1	∙∙
28	Ethanol	0.02 %	1 h	TCF12	∙∙
29	Ethanol	0.02 %	1 h	USF1	∙∙
30	None	None	None	CTCF	∙∙
31	None	None	None	PBX3	∙∙
32	None	None	None	RAD21	∙∙
33	None	None	None	TEAD4	∙∙

**Table 2:** Target proteins of the used datasets regarding treatments with Dexamethasone (Dex), or Ethanol (EtOH), or with no treatment (None)

#	Target protein	Antibody target	Associated disease (main)	Dex	EtOH	None
1	Activating Transcription Factor 3	ATF3	Hodgkin’s lymphoma [38]		✓	
2	B-cell lymphoma 3	BCL3	Lymphoma and chronic lymphocytic leukemia [39]		✓	
3	Transcriptional repressor CTCF	CTCF	Regulation of chromatin architecture [40]		✓	✓
4	E1A binding protein p300	EP300	Rubinstein-Taybi syndrome [41]		✓	
5	Forkhead box protein A1	FOXA1	Estrogen receptor *α* (ER *α*) breast cancer [42]	✓		
6	GA-binding protein alpha chain	GABPA	Down syndrome [43]		✓	
7	Transcription factor jun-B	JUNB	Myeloproliferative disorder [44]	✓		
8	Transcription factor jun-D	JUND	Adult-T cell leukaemia [45]		✓	
9	Glucocorticoid receptor	NR3C1	Glucocorticoid resistance syndrome [46]	✓		
10	Pre-B-cell leukemia transcription factor 3	PBX3	Pilocytic astrocytoma [47]			✓
11	DNA-directed RNA polymerase II subunit RPB1	POLR2A	UV-sensitive syndrome [48]	✓	✓	
12	Double-strand-break repair protein rad21 homolog	RAD21	Cornelia de Lange syndrome [49]			✓
13	RE1-silencing transcription factor	REST	Wilms tumor [50]		✓	
14	Paired amphipathic helix protein Sin3a	SIN3A	Chromosome 15q24 microdeletion syndrome [51]		✓	
15	Homeobox protein SIX5	SIX5	Branchio-oto-renal syndrome [52]		✓	
16	Transcription initiation factor TFIID subunit 1	TAF1	X-linked dystonia-parkinsonism [53]		✓	
17	Transcription factor 12	TCF12	Extraskeletal myxoid chondrosarcoma [54]		✓	
18	Transcriptional enhancer factor TEF-3	TEAD4	Narcolepsy [55]			✓
19	Upstream stimulatory factor 1	USF1	Hyperlipidemia [56]	✓	✓	

### Data preparation

Each dataset consists of 2-3 (isogenous) replicates. The replicates were comparatively evaluated using the Multiple Sample Peak Calling (MSPC) method [32], which locally lowers the minimum significance required to accept repeated evidences across replicates. We used MuSERA [33], a graphical implementation of the MSPC method, to combine multiple replicates of DNA-binding enriched region (i.e., called peak) samples of a dataset into a single sample without loosing or overestimating the significance of the called peak regions.

**Fig. 6:**
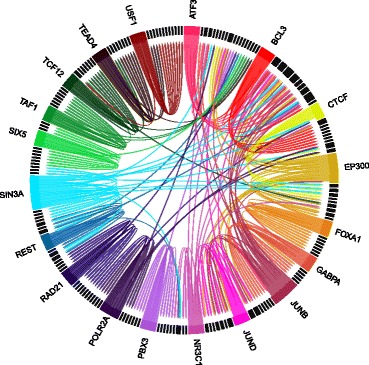
Protein-protein interaction. The labeled proteins are the considered target proteins summarized on Table 2, and the unlabeled proteins are the proteins that interact with at least one of the target proteins

Each of the considered datasets has a target protein (summarized on Table 2). As the function of proteins tends to be regulated by other proteins (cf. interactomics), we used STRING [34] to search for protein-protein interactions for each of the dataset target proteins. We found 163 proteins that interact with at least one of the dataset target proteins (see Fig. 6). We focused on these 182 proteins (i.e., 19 target proteins, and 163 proteins with which the target proteins interact).

As reference genomic regions, we used RefSeq [35] human gene annotations downloaded from Ensembl [36], focusing on those genes regarding the selected proteins based on gene name; we found 171 of them.

**Fig. 7:**
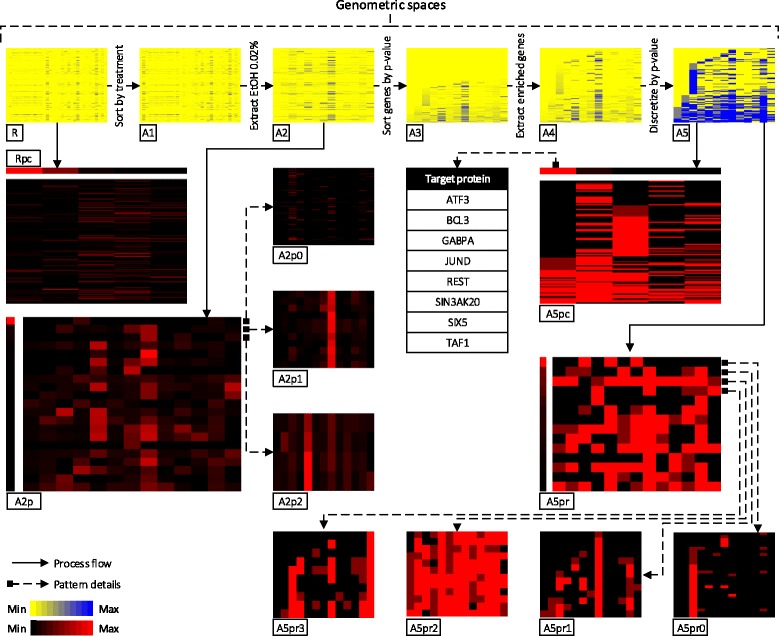
Exploring the effect of treatment with Ethanol 0.02% on gene-binding enrichment of several proteins. Heatmaps are displayed in thumbnail size (since at this resolution the labels of rows and columns would not be readable, we removed them and provide full size labeled heatmaps at http://www.bioinformatics.deib. polimi.it/GeMSE/). The heatmaps in yellow-blue and black-red color scale represent the genometric spaces generated in the GeMSE STT during the exploration, and the extracted patterns, respectively, for the binding enrichment significance. Panel Rpc shows the patterns of gene-protein binding enrichment significance found for the samples/treatments-proteins in the initial genometric space R. Panels A2p0, A2p1, and A2p2 plot the vectors (genes) respectively forming the first, second, and third most common patterns of those found (shown on panel A2p) for the genometric space A2. The table explains the vectors (samples) forming the most common pattern on panel A5pc in terms of the“Antibody target” attribute of the sample metadata. Panels A5pr0, A5pr1, A5pr2, and A2pr3 plot the vectors (genes) orderly forming the first four most common patterns of those found (shown on panel A5pr) for the genometric space A5

In GeMSE we loaded a reference sample with the considered genes, and the 33 replicate-combined ChIP-seq experiment samples obtained; thus, we mapped every DNA-protein binding enriched region in each of the latter samples on the considered genes (see flowchart and panel b on Fig. 1), and computed aggregate values of the attributes associated with the regions in each ChIP-seq sample that overlap each gene (i.e., region counts, averages of region *p*-values). In so doing, we built a genometric space R with 171 rows (genes) and 33 columns (samples/conditions) (see panel R on Fig. 7), which we fully explored and interactively analyzed by taking advantage of GeMSE.

### Data exploration

As an example, in our scenario GeMSE can be used to search for experiment samples with similar profiles of gene-protein binding enrichment significance. GeMSE can extract patterns of such profiles in the considered genometric space, leveraging on the following data transformation:

$$R^{\prime} = \texttt{Cluster} \; (\texttt{C}, \texttt{AVERAGE}, {\tt EU}) \; R $$

In our case, GeMSE suggests the existence of 5 of such patterns (see panel Rpc on Fig. 7), and supports their explanation based on the metadata of samples sharing the same pattern (see Table 3). Referring to Table 3, all 10 jun-B samples with Dex 100 nM treatment for various durations are grouped together in pattern P-1, as well as both samples targeting POLR2A are in pattern P-2. These are interesting, yet expected, results that GeMSE highlights; answers to several other questions can be discovered through GeMSE. In the following subsections, we show how to discover more interesting aspects of the data by interactively exploring them taking advantage of the easy-to-use graphical interface for interactive analytics of GeMSE.

**Table 3:** Excerpt of metadata aggregation for the five patterns of gene-protein binding enrichment significance that correspond to the root of the STT of the performed data exploration described in Fig. 7 (see panel Rpc on Fig. 7)

Attribute	Value	P-0	P-1	P-2	P-3	P-4
Antibody target	POLR2A	0	0	2	0	0
Treatment	Dex 100 nM 30 m	0	1	0	0	0
Treatment	Dex 100 nM 0 h	0	1	0	0	0
Treatment	Dex 100 nM 1 h	0	1	0	0	0
Treatment	Dex 100 nM 2 h	0	1	0	0	0
Treatment	Dex 100 nM 3 h	0	1	0	0	0
Treatment	Dex 100 nM 4 h	0	1	0	0	0
Treatment	Dex 100 nM 5 h	0	1	0	0	0
Treatment	Dex 100 nM 7 h	0	1	0	0	0
Treatment	Dex 100 nM 8 h	0	1	0	0	0
Treatment	Dex 100 nM 10 h	0	1	0	0	0

### Effects of Ethanol treatment

In this subsection, we show how GeMSE can help in determining the effects of ethanol treatment on gene-protein binding enrichment profiles.

A number of considered input samples regard studying the effect of the treatment with Ethanol 0.02 % on the DNA-binding enrichment profile of various proteins. To focus on these samples, in GeMSE we first sort data as follows (see panel A1 on Fig. 7):

$$A1 = \texttt{Sort} \; (\texttt{C}, \texttt{ASCENDING}, \texttt{Treatment}) \; R $$

By setting column labels of the heatmap to “Treatment”, and looking at the *heatmap* or at the *grid view* (a tabular representation of a genometric space in GeMSE) of the result, we see that obtained columns 16-28 represent samples with EtOH 0.02 % treatment; we extract these columns as follows (see panel A2 on Fig. 7):

$$A2 = \texttt{Extract} \; ([16, 29), [0, 171)) \; A1 $$

Then, we search for patterns of gene-protein binding enrichment significance only on the extracted samples with EtOH 0.02 % treatment; this can be done by clustering the obtained genometric space A2 by rows/genes (instead of by columns/samples, as in the initial example). Leveraging on the following data transformation:

$$A2^{\prime} = \texttt{Cluster} \; (\texttt{R}, \texttt{AVERAGE}, {\tt EU}) \; A2 $$

**Fig. 8:**
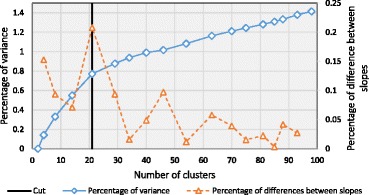
Application of the Elbow method for finding the optimal number of clusters on A2 genometric space of Fig. 7. Based on this method, the optimal number of clusters is 21

GeMSE suggests 21 patterns (see Fig. 8 for Elbow method data), each representing a group of genes with similar profiles of gene-protein binding enrichment significance for the extracted EtOH 0.02 % treatment samples (see panel A2p on Fig. 7).

GeMSE allows further exploration of each of the extracted patterns, by expanding a pattern to the individual elements it groups (in this case, genes) and visualizing on a heatmap the values of the element associated attribute considered (in this case, binding enrichment significance *p*-value for each of the grouped genes and each evaluated sample). For instance, the three upper most patterns in panel A2p on Fig. 7 are expanded to the contributing genes and plotted on panels A2p0, A2p1, and A2p2 on Fig. 7. A pattern can also be described by using the metadata of the elements it groups; e.g., the left most pattern in panel A5pc on Fig. 7 is described in the table on Fig. 7 using sample metadata. Additionally, GeMSE allows using any of the numerical attributes associated with the pattern elements (e.g., in our case, *p*-value, q-value, region count) for visualization of the individual components of a pattern. This allows assessing patterns based on various quantifying attributes.

Popularity of a pattern within a dataset (i.e., number of dataset elements sharing the pattern) can also be easily observed. Combining such observation with the intensity of the attribute values associated with the pattern elements can provide useful support for further evaluations. For example, the upper most pattern in panel A2p on Fig. 7 is very common (117 out of 171 genes share it; see panel A2p0 on Fig. 7). Yet, it is formed by genes with no or weak protein binding enrichment across all samples; thus, we may not be interested in such pattern. We may also exclude the genes with low or no protein binding enrichment on all samples; such gene filtering can be done as follows.

We can first sort the rows/genes of the genometric space in ascending order (instead of sorting by columns/samples, as previously done) as follows (see panel A3 on Fig. 7):

$$A3 = \texttt{Sort} \; (\texttt{R}, \texttt{ASCENDING}, p-\text{value}) \; A2 $$

Then, using a *grid view* we look at the sorted genometric space A3 and identify the row *r* (with *r*=72, in our case) as the first row/gene with protein binding enrichment in at least one of the samples (i.e., all the genes at rows before *r* have protein binding enrichment in neither of the samples, and all the genes on and after *r* have protein binding enrichment at least in one of the samples). Then, we extract rows from *r* to the last row of the ordered genometric space A3 as follows (see panel A4 on Fig. 7):

$$A4 = \texttt{Extract} \; ([0, 13), [72, 171)) \; A3 $$

Even after removing genes with no protein binding enrichment, some of the remaining genes may have a low significant protein binding enrichment, while some others may be highly significantly enriched. It may then be useful to discretize gene-protein binding enrichment significance as *weak*, *middle*, or *strong*. Given the nature of significance *p*-values, it may be worth grouping together data with *p*-values 1·e^−12^ (significant) and 1·e^−200^ (very significant), rather than grouping data with *p*-values 1·e^−12^ (significant) and 1·e^−4^ (low significant), as it would probably occur by *p*-value clustering. This is obtained by the Discretize transformation, applied on A4 as it follows, where the *p*-values 40 and 80 are in −10· log10 format as in the A4 data (see panel A5 on Fig. 7):

$$A5 = \texttt{Discretize} \; ([0, 13), [0, 99), [40, 0, 1], [80, 1, 2]) \; A4 $$

On the discretized genometric space A5, we can search for genes with similar pattern of protein binding enrichment significance, and find the patterns in panel A5pr on Fig. 7. We note that:

1. The most common pattern found (see panel A5pr0 on Fig. 7) includes genes with significant protein binding enrichment in samples targeting CTCF or REST proteins (row number 19 and 23 on Table 1).
2. The second most common pattern (see panel A5pr1 on Fig. 7) includes genes that have mostly significant enrichment of POLR2A binding (sample in row number 24 on Table 1).
3. The third most common pattern (see panel A5pr2 on Fig. 7) includes genes with significant protein binding enrichment in most of the samples.
4. The forth most common pattern (see panel A5pr3 on Fig. 7) includes the set of genes with mostly significant enrichment of USF1 binding (sample in row 29 on Table 1).

Then, on the discretized genometric space A5, we can also search for samples with similar pattern of protein binding enrichment significance. Based on the GeMSE suggested number of clusters, we find 5 patterns (see panel A5pc on Fig. 7), with one of them in common among 8 out of 13 samples with EtOH 0.02 % treatment. We use GeMSE to explore this pattern, and choose to see the values of the “Antibody target” metadata attribute of the samples with this pattern; this operation lists all the target proteins of such samples (see the table on Fig. 7), which include proteins SIN3A and REST. This finding might have several interpretations; for instance, an explanation could be that the REST transcription factor is known to repress transcription by recruiting the corepressor SIN3A [37].

### Effects of Dexamethasone

Here, we show how various doses of Dexamethasone treatment affect gene-binding enrichment of the NR3C1 protein, by using GeMSE on the considered data. Solving this problem requires a data exploration procedure different from the previously performed one, starting from the genometric space A1 obtained after the sort by treatment operation at the beginning of the previously described data exploration, and ready available in the GeMSE STT (see Fig. 3). As our considered data include three samples targeting the NR3C1 protein and regarding Dexamethasone treatment with 500 pM, 5 nM and 50 nM dose, respectively, first we want to extract their corresponding columns from A1. After looking at the A1 heatmap and identifying the required columns as columns 13-15 in A1, we extract them as follows:

$$B1 = \texttt{Extract} \; ([13, 16), [0, 171)) \; A1 $$

**Fig. 9:**
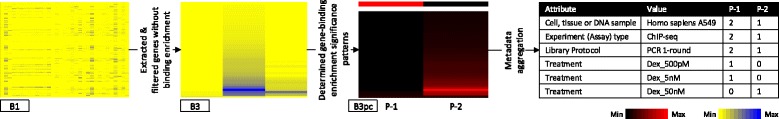
Exploring the effect of treatment with various doses of Dexamethasone on the enrichment of the gene-binding of the NR3C1 protein. From the initial panel B1, three columns corresponding to samples regarding treatments with different Dexamethasone doses (500 pM, 5 nM and 50 nM) are extracted, and from them genes without binding enrichment are removed (panel B3); finally, patterns of gene-binding enrichment significance are extracted in panel B3pc. Metadata aggregations of the two identified patterns are shown in the table. Heatmaps at full size and with their row/column labels are available at http://www.bioinformatics.deib.polimi.it/GeMSE/

Then, we remove genes without protein binding enrichment as done in previous exploration, i.e., through gene sorting by enrichment *p*-value, visual inspection of the heatmap of the sorted genometric space B2 obtained, identification of the row *r* in B2 corresponding to the first gene with NR3C1 binding enrichment in at least one of the samples (i.e., *r*=130, in our case), and extraction of the rows from *r* to the last row of the ordered genometric space B2 (see panel B3 on Fig. 9).

Then, we search for patterns of gene-binding enrichment significance across the three samples. GeMSE suggests 2 patterns of enrichment significance for the gene-binding of the NR3C1 protein (see panel B3pc on Fig. 9), where the binding enrichment significance for the treatment with 50 nM of Dex are in a separate group from the significance regarding the treatments with lower doses of Dex, i.e., 5 nM and 500 pM. This can be seen also from the metadata aggregation table that GeMSE provides (see an excerpt of it in the table on Fig. 9).

## Conclusions

The availability of huge, well-curated and open repositories of processed genomic datasets motivates our efforts in designing and implementing new data exploration abstractions, so as to facilitate effective biological knowledge discovery through interactive analytics.

Thanks to the notion of genometric space, our GeMSE tool at the same time rises the expressive power of user-data interaction and lowers the complexity of data exploration, making it available to nonprogrammers. The tool supports a trial-and-error approach that can be very useful for both defining the appropriate knowledge extraction pipelines and exploring alternative hypotheses, making GeMSE a relevant interactive analytics application.

GeMSE effectively provides the tracing of data exploration steps through a state-transition diagram, whose states, which represent exploration step results, are all accessible at any time; this is obtained thanks to an efficient algorithm for state-transition caching and reconstruction implemented in the tool. Evolution of the data exploration occurs by means of state transitions which embody genometric space transformations.

GeMSE effective application and practical usefulness is demonstrated through significant use cases of biological interest.

## Availability and requirements

**Project name:** GeMSE

**Project homepage:** http://www.bioinformatics.deib.polimi.it/GeMSE/

**Project source code and discussions and issues page:** https://github.com/Genometric/GeMSE

**License:** GPL v3.0

**Operating system(s):** platform independent (tested on Microsoft Windows 10, macOS Sierra, and Ubuntu 16).

**Programming language:** Java

**Other requirements:** if chosen to connect with R, GeMSE requires R installation with gplots, ape, and hclust packages installed.

**Tutorial and example data:** available at project homepage.

## Abbreviations

BED: Browser extensible data
CDS: Coding sequence
CSV: Comma-separated value
Dex: Dexamethasone
DNA: Deoxyribo nucleic acid
ENCODE: Encyclopedia of DNA elements
EtOH: Ethanol
GeMSE: GenoMetric space explorer
GMQL: GenoMetric query language
GTF: General transfer format
IA: Interactive analytics
MSPC: Multiple sample peak calling
MuSERA: Multiple sample enrichment region assessment
NGS: Next generation sequencing
STRING: Search tool for the retrieval of interacting genes
STT: State-transition tree
TCGA: The cancer genome atlas
UCSC: University of california santa cruz
UTR: UnTranslated region
